# Expression of PD-L1 through evolution phase from pre-invasive to invasive lung adenocarcinoma

**DOI:** 10.1186/s12890-023-02310-0

**Published:** 2023-01-16

**Authors:** Jiebai Zhou, Huo Lin, Zheng Ni, Rongkui Luo, Dawei Yang, Mingxiang Feng, Yong Zhang

**Affiliations:** 1 Department of Pulmonary and Critical Care Medicine, Zhongshan Hospital, Fudan University, Shanghai, China; 2 Department of Pulmonary and Critical Care Medicine, Shishi County Hospital, Shishi, Fujian China; 3 Department of Pathology, Zhongshan Hospital, Fudan University, Shanghai, China; 4 Department of Thoracic Surgery, Zhongshan Hospital, Fudan University, Shanghai, China

**Keywords:** Lung cancer, Early-stage, PD-L1 expression, Adenocarcinoma evolution

## Abstract

**Background:**

This study evaluated programmed cell death-ligand 1 (PD-L1) expression from pre-invasive adenocarcinoma to invasive lung adenocarcinoma, aimed to investigate the potential association of PD-L1 pathway with lung adenocarcinoma early evolution.

**Methods:**

We evaluated PD-L1 expression in 1123 resected lung specimens of adenocarcinoma in situ (AIS), minimally invasive adenocarcinoma (MIA) and invasive adenocarcinoma (IAC) of stage IA1–IA3. PD-L1 expression was defined based on the proportion of stained tumor cells using the tumor proportion score:  < 1% (negative),  ≥ 1% (positive) and  ≥ 50% (strongly positive). Correlations between PD-L1 expression and T stage, pathological subtype, adenocarcinoma grade, spread through air space (STAS), vascular invasion, lymphatic invasion and driven genes were analyzed.

**Results:**

There was almost no PD-L1 expression in AIS or MIA. However, PD-L1 expression was correlated with invasiveness of lung adenocarcinoma. The percentages of PD-L1 positive in IA1–IA3 were 7.22%, 11.29%, and 14.20%, respectively. The strongly positive rates of PD-L1 were 0.38%, 1.64%, and 3.70% in IA1–IA3, respectively. PD-L1 expression and positive rate were also associated with poor pathological subtype and poor biological behavior, such as adenocarcinoma Grade 3, micropapillary or solid dominant subtype, STAS and vascular invasion. Finally, PD-L1 positive rate seems also corrected with driven gene *ALK, ROS-1* and *KRAS*.

**Conclusions:**

PD-L1 expression was positively correlated with the emergence of invasiveness and poor pathological subtype or biological behavior of early-stage lung adenocarcinoma. PD-L1 pathway may be involved in the early evolution of lung adenocarcinoma from AIS to IAC.

## Background

Overexpression of programmed cell death‐ligand 1 (PD‐L1) on tumor cells is one primary mechanism of immune escape in cancer [1]. PD-L1 inhibits apoptosis of the tumor cell, promotes peripheral T effector cell exhaustion and conversion of T effector cells to regulatory T cells through its interaction with programmed cell death‐1 (PD‐1) on T cells [2, 3]. Therapeutic antibodies targeting the PD-1/PD-L1 axis have been developed for clinical application, demonstrating remarkable clinical responses in patients with advanced lung cancer [2]. Significantly, high PD‐L1 expression is associated with a better clinical response to PD‐1/PD‐L1 antibodies [3–5]. PD-L1 immunohistochemical detection has been established as a standard biomarker for the activity of the PD‐1/PD‐L1 pathway [6, 7].

Adenocarcinoma in situ (AIS) was pre-invasive lesion, according to the 8th lung cancer TNM stage system [8]. AIS, minimally invasive adenocarcinoma (MIA) and invasive adenocarcinoma (IAC) were considered three different evolution phase of early-stage lung adenocarcinoma according to invasive degree [9]. Former small sample studies showed PD-L1 expression were negative in AIS or MIA and may be positive in stage IA lung adenocarcinoma [10]. Another study indicated the increasing PD-L1 expression in solid nodule group than in the ground-glass opacity (GGO) group [11]. There was also one study showed PD-L1 expression was associated with immune cell infiltration within stage IA lung adenocarcinoma [12]. Besides, PD-L1 expression may predicts poor prognosis in the recurrence of resected stage IA lung cancer [13]. These data indicated PD-1/PD-L1 pathway may be involved in early-stage invasive lung adenocarcinoma.

This study analyzed the PD‐L1 expression in early-stage lung adenocarcinoma, including AIS, MIA, and IAC (stage IA1–IA3) in a cohort of 1,123 tumors. We investigated the association of PD-L1 expression with T stage, lung adenocarcinoma pathological grade, which was aroused by the International Association for the Study of Lung Cancer (IASLC) in 2020 [14], pathological dominant subtype, spread through air space (STAS), vascular invasion, lymphatic invasion and seven driven genes, in the hope of comprehensively demonstrating PD-L1 expression status in stage 0-IA lung adenocarcinoma and add the evidence of potential involvement of PD-1/PD-L1 pathway in the evolution of lung adenocarcinoma from AIS to IAC.

## Methods

### Patient selection

**Fig. 1:**
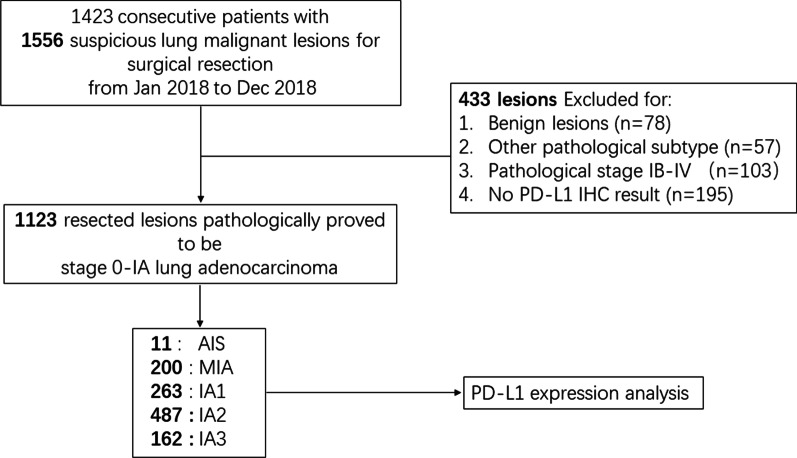
Consort diagram of patient selection. *IHC* Immunohistochemistry, *AIS* Adenocarcinoma in situ, *MIA* Minimally invasive adenocarcinoma, *PD-L1* Program death-1 ligand1

We enrolled consecutive patients with suspicious lung malignant lesions for surgical resection at Zhongshan Hospital, Fudan University, Shanghai, China from Jan 2018 to Dec 2018. The cases of benign lesions, other pathological subtype, pathological stage IB-IV and with no PD-L1 IHC result were excluded after surgery. Eventually, a total of 1123 lesions pathologically proved to be stage 0–IA lung adenocarcinoma from AIS, MIA to IAC (stage IA1–IA3) were enrolled in the study. A consort diagram of patient selection was shown as Fig. 1.

### Assessment of PD-L1 expression

PD-L1 expression of DAKO 28-8 pharmDx assay was evaluated by SP-28-8 antibody (Abcam Corp., Cambridge, UK) with immunohistochemistry method by pathologists at Zhongshan Hospital. In brief, rabbit monoclonal anti-PD-L1 was used in the detection of PD-L1 protein in FFPE tissue with the EnVision FLEX visualization system on Autostainer Link 48. Staining protocol was provided by the package of the DAKO interpretation guide. All cases had accompanying controls, H&E-stained patient slide, negative reagent control-stained patient slide, and a DAKO PD-L1 28-8-stained patient slide. PD-L1 positivity were evaluated using a tumor cell expression (TC) scoring method, where TC = (number of PD-L1-stained tumor cells/total tumor cells) × 100: < 1% (negative),  ≥ 1% (positive), and  ≥ 50% (strongly positive) for the DAKO PD-L1 28–8 pharmDx assay.

### Histologic analysis

The pathological results in this article were evaluated by pathologists at Zhongshan Hospital. The diagnosis of early-stage lung adenocarcinoma was determined by the histopathological examination of resected specimens. The lesions were classified as AIS, MIA, or IAC (stage IA1–IA3) according to the lung cancer 8th TNM stage system, which was aroused by IASLC, the American Thoracic Society (ATS), the European Respiratory Society (ERS), and the World Health Organization (WHO) classifications of lung neoplasms. AIS and MIA were both ground glass opacity (GGO) dominant in CT scan. AIS is a non-invasive lesion, MIA has an invasive component of less than 0.5 cm, and IACs of stages IA1, IA2, and IA3 have an invasive part of 0.5 to 1 cm, 1 to 2 cm, and 2 to 3 cm, respectively.

Adenocarcinoma histologic grade was characterized according to the IASLC system as follows: grade 1, lepidic-predominant tumors with no or  < 20% high-grade patterns (solid, micropapillary, and/or complex glandular patterns); grade 2, acinar- or papillary-predominant tumors with no or  < 20% high-grade patterns; and grade 3, any tumor with  ≥ 20% high-grade patterns [14]. STAS status was defined as the micropapillary clusters, solid nests, or single cells of tumor that exist in the air spaces of the surrounding lung parenchyma beyond the edge of the main tumor [15]. Vascular and lymphatic invasion of each tumor were also evaluated.

### Driven gene detection

Genotypic analysis was performed in all of our cases by the amplification refractory mutation system (ARMS) PCR method with Pan Lung Cancer PCR Panel (AmoyDx Corp., XM, China) according to standard protocol. Seven driver oncogene (*EGFR*, *KRAS*, *ALK*, *ROS-1*, *BRAF, HER2* and *RET*) mutations in lung adenocarcinoma were analyzed in all the resected tissue. The driven gene results in this article were also evaluated by pathologists at Zhongshan Hospital.

### Statistical analysis

All statistical analyses were performed by SPSS 19.0 (IBM Corp., Armonk, NY, USA). The count data are presented as the number and percentage. Fisher's exact test was utilized and *p* value less than 0.05 was considered as significance level.

## Results

### Clinical characteristics of the patients

There was a total of 1,123 tumors included in this study. Eleven tumors were confirmed to be AIS, and 200 were confirmed to be MIA. There were also 912 cases in IAC groups (Table 1). Clinical information including age, sex and smoking history were collected. The group of Age  ≥ 65 years old seems be more possibly associated with IAC with statistical significance (*p* < 0.001). However, Sex and smoking history seem has no difference in the three group of AIS, MIA and IAC.

**Table 1:** Patient information and association of PD-L1 expression with AIS, MIA and IAC

	AIS (%)		MIA (%)		IAC (%)		*p* value	Total
*Age (year)*								
≥ 65	2	0.60	26	7.81	305	91.59	< 0.001	333
< 65	9	1.14	174	22.03	607	76.84		790
*Sex*								
Male	4	1.05	57	14.96	320	83.99	0.201	381
Female	7	0.94	143	19.27	592	79.78		742
*Smoking history*								
Yes	2	1.54	15	11.54	113	86.92	0.116	130
No	9	0.91	185	18.63	799	80.46		993

### PD-L1 expression in AIS, MIA and stage IA1–IA3 lung adenocarcinoma

**Fig. 2:**
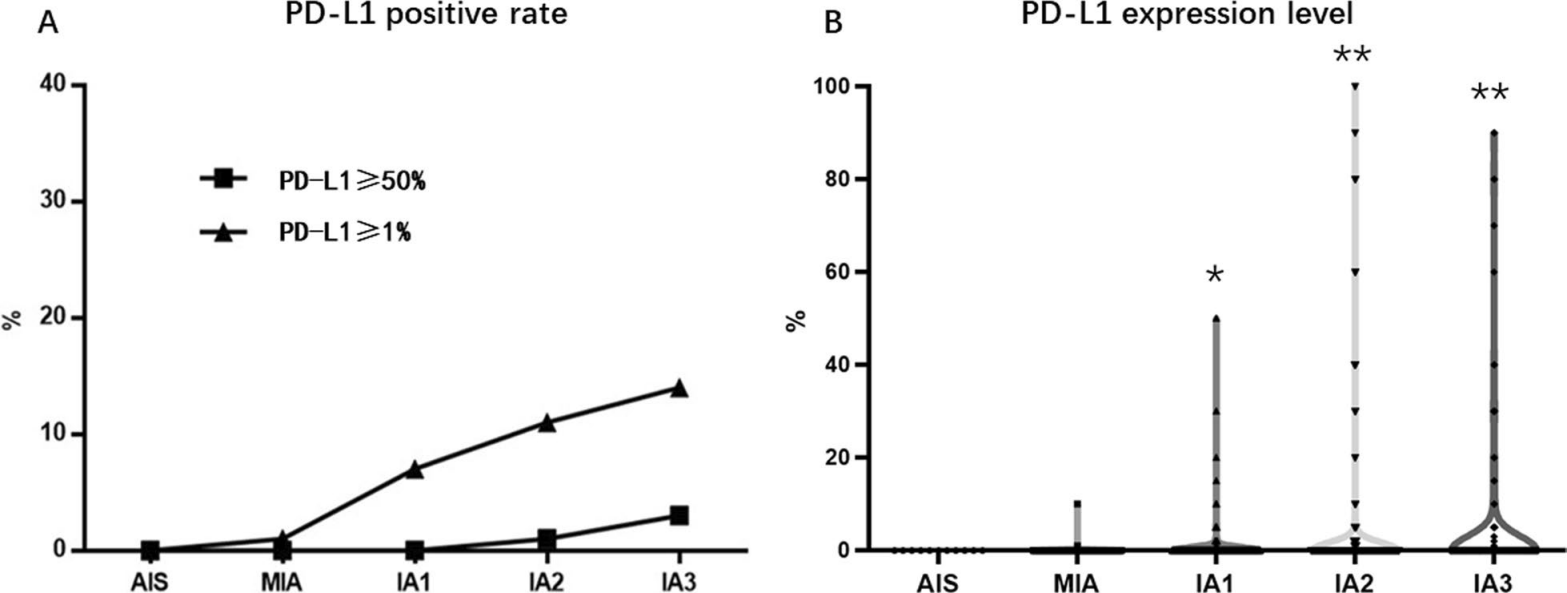
PD-L1 positive rate and expression level in AIS, MIA and IAC of stage IA1–IA3 **A** The percentages of PD-L1-positive (≥ 1%) samples and PD-L1-strongly-positive (≥ 50%) samples increased according to lung adenocarcinoma invasiveness from AIS to IAC (IA1–IA3) (*p* < 0.001). **B** The PD-L1 expression level increased according to lung adenocarcinoma invasiveness from AIS to IAC (IA1–IA3) (*p < 0.05, **p < 0.01)

**Fig. 3:**
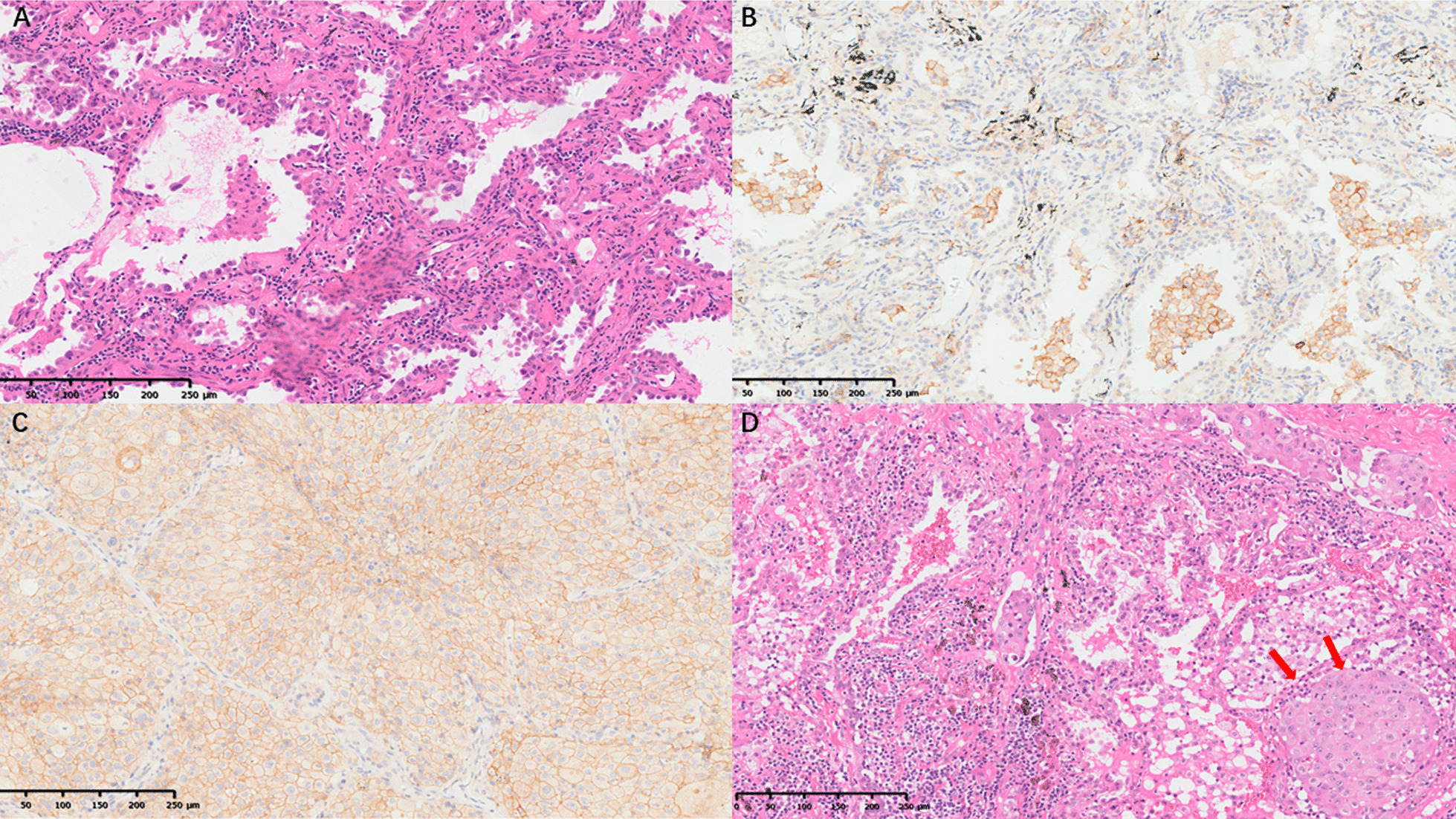
Typical pathological pictures (100x) of case 1: adenocarcinoma in situ (AIS) **A** hematoxylin–eosin (HE) staining showed the lepidic growth of tumor cells with **B** PD-L1 (SP-28-8) staining negative (< 1%); case 2: Invasive adenocarcinoma (IAC) stage IA3 of solid dominant subtype **C** PD-L1 (SP-28-8) staining strongly positive (80%) and **D** spread through air space (STAS) of the solid nest (red arrow) beyond tumor edge

PD-L1 positive rate in early-stage lung adenocarcinoma from AIS, MIA to IAC stage IA1–IA3 was shown in Table 2. PD-L1 expression was almost negative in AIS and MIA. There was no PD-L1 positive case in AIS. Two (1%) of the MIA cases showed positive PD-L1 expression. Interestingly, PD-L1 expression increased with the invasiveness of lung adenocarcinoma. The percentages of PD-L1 positive samples in stage IA1–IA3 lung adenocarcinoma were 7.22%, 11.29%, and 14.20%, respectively. The percentages of PD-L1 strongly positive samples also increased according to invasiveness, which were 0.38%, 1.64%, and 3.70% in stage IA1–IA3, respectively (Fig. 2A). The PD-L1 expression level also increases according to lung adenocarcinoma invasiveness (IA1–IA3) (Fig. 2B). A typical pathology picture of AIS with negative PD-L1 expression was shown in Fig. 3A and B.

**Table 2:** PD-L1 positive rate in early-stage lung adenocarcinoma from AIS, MIA to IAC stage IA1–IA3

	PDL1 (−) (%)		PD-L1≥1%		*p* value	PD-L1≥50%		*p* value	Total
AIS	11	100.00	0	0.00	< 0.001	0	0.00	0.012	11
MIA	198	99.00	2	1.00		0	0.00		200
IA1	244	92.78	19	7.22		1	0.38		263
IA2	432	88.71	55	11.29		8	1.64		487
IA3	139	85.80	23	14.20		6	3.70		162
IA (Grade 1)	20	86.96	3	13.04	< 0.001	0	0.00	< 0.001	23
IA (Grade 2)	739	91.23	71	8.77		9	1.11		810
IA (Grade 3)	56	70.89	23	29.11		6	7.59		79
Lepidic dominant	83	92.22	7	7.78	< 0.001	0	0.00	< 0.001	90
Acinar dominant	660	90.91	66	9.09		7	0.96		726
Papillary dominant	36	92.31	3	7.69		0	0.00		39
Micropapillary dominant	3	60.00	2	40.00		0	0.00		5
Solid dominant	10	45.45	12	54.55		5	22.73		22
Invasive mucinous	25	89.29	3	10.71		1	3.57		28
STAS(+)	37	75.51	12	24.49	< 0.001	3	6.12	0.017	49
STAS(-)	987	91.90	87	8.10		12	1.12		1074
Vascular invasion (+)	12	66.67	6	33.33	< 0.001	3	16.67	0.001	18
Vascular invasion (-)	1012	91.58	93	8.42		12	1.09		1105
Lymphatic invasion (+)	1	100.00	0	0.00	N/A	0	0.00	N/A	1
Lymphatic invasion (-)	1023	91.10	99	9.68		15	1.46		1122

*AIS* Adenocarcinoma in situ; *MIA* Minimally invasive adenocarcinoma; *IAC* Invasive adenocarcinoma; *STAS* Spread through air space, *N/A* Not applicable

### PD-L1 expression in lung adenocarcinoma grade and dominant subtype

**Fig. 4:**
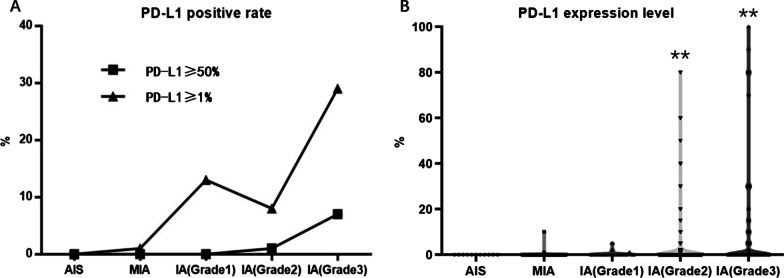
PD-L1 positive rate and expression level in AIS, MIA and pathological grade 1–3. **A** The percentages of PD-L1-positive (≥ 1%) samples and PD-L1-strongly-positive (≥ 50%) samples increased according to lung adenocarcinoma pathological grade 1–3 (*p* < 0.001). **B** PD-L1 expression level increased according to lung adenocarcinoma pathological grade 1–3 (**p < 0.01)

PD-L1 expression was positively correlated with lung adenocarcinoma Grade 3 (Table 2). The percentages of PD-L1 positive samples in stage IA of Grade1 and Grade2 were 13.04% and 8.77%, respectively. The difference showed no statistical significance. However, the rate of PD-L1-positive samples in stage IA of Grade3 remarkably increased to 29.11% (*p* < 0.001). The percentages of PD-L1 strongly positive samples increased according to lung adenocarcinoma pathological grade, which were 0%, 1.11%, and 7.59% in Grade 1–3, respectively (*p* < 0.001) (Fig. 4A). The PD-L1 expression level also increase according to lung adenocarcinoma pathological Grade (Grade1–Grade3) (Fig. 4B). A typical pathological figure showing stage IA3 adenocarcinoma of grade 3 (solid subtype dominant) of PD-L1 strongly positive (80%) sample was displayed in Fig. 3C.

**Fig. 5:**
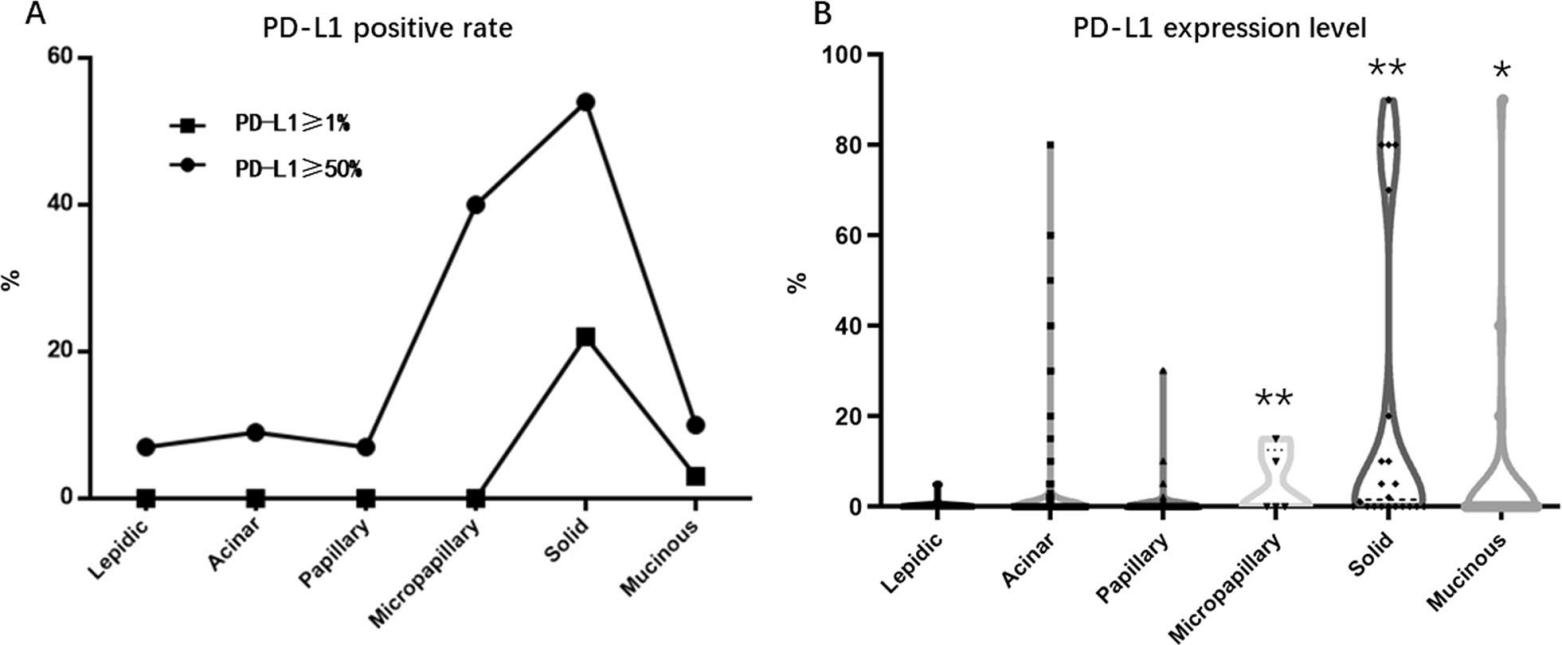
PD-L1 expression of different adenocarcinoma subtype. **A** The percentages of PD-L1-positive (≥ 1%) samples and PD-L1-strongly-positive (≥ 50%) samples increased in solid dominant subtype, **B** PD-L1 expression level increased in micropapillary, solid and mucinous dominant subtype (*p < 0.05, **p < 0.01),

There was an evident higher PD-L1 expression in IAC of solid subtype dominant than other subtypes. PD-L1 positive rate in the solid subtype dominant cases was 54.55%, which was much higher than other subtypes (*p* < 0.001). The percentage of PD-L1 strongly positive samples was also higher in solid cases (22.73%, *p* < 0.001) (Fig. 5A). PD-L1 expression level was also higher in solid, micropapillary and mucinous dominant subtype compared with lepidic dominant subtype (Fig. 5B).

### Association between PD-L1 expression and STAS or vascular invasion status

PD-L1 expression was associated with STAS and vascular invasion status of stage IA lung adenocarcinoma. PD-L1 positive rate in the STAS positive group was 24.49%, which was much higher than that in the STAS negative group (8.10%) (*p* < 0.05). The percentage of PD-L1 strongly positive samples was also higher in STAS ( +) cases than that in the STAS (−) group (6.12% vs. 1.12%, *p* < 0.001) (Table 2). A typical pathological figure showing STAS status in the case of stage IA3 adenocarcinoma of a solid predominant subtype of PD-L1 strongly positive (80%) sample is displayed in (Fig. 3D). Besides, PD-L1 positive rate in the vascular invasion ( +) group was 33.33%, which was also much higher than that in the vascular invasion (−) group (8.42%) (*p* < 0.05). The percentage of PD-L1 strongly positive samples was also higher in vascular invasion ( +) cases than that in the vascular invasion (−) group (16.67% vs. 1.09%, *p* < 0.001) (Table 2). In addition, the association of PD-L1 expression and lymphatic invasion were not applicable due to insufficient cases.

### Association between PD-L1 expression and driven genes

The increased PD-L1 positive rate seemed to be associated with mutations in driver genes of *KRAS*, *ROS1*, and *ALK*. PD-L1 positive rate increased in the *KRAS* (32.00%) and *ROS-1*(44.44%) mutated group (*p* < 0.01), and PD-L1 strongly positive rate increased in the *ALK* (8%) mutated group (Table 3). However, there seemed to be no connection between PD-L1 expression and *EGFR, RET, HER2 and BRAF* mutation in our data.

**Table 3:** PD-L1 expression in stage 0–IA lung adenocarcinoma with driven genes (*p*: vs Driven gene negative)

	PDL1 (−) (%)		PD-L1 ≥ 1%		*p* value	PD-L1 ≥ 50%		*p* value	Total
EGFR ( +)	609	91.72	56	8.43	0.795	5	0.75	0.353	665
ALK ( +)	20	80.00	5	20.00	0.068	2	8.00	0.046	25
ROS-1 ( +)	5	55.56	4	44.44	0.006	1	11.11	N/A	9
Kras ( +)	17	68.00	8	32.00	0.001	1	4.00	N/A	25
RET ( +)	10	90.91	1	9.09	N/A	0	0.00	N/A	11
HER2 ( +)	23	100.00	0	0.00	N/A	0	0.00	N/A	23
BRAF ( +)	3	100.00	0	0.00	N/A	0	0.00	N/A	3
Driven gene (−)	337	92.84	26	7.16		6	1.65		363
Total	1024	91.18	99	8.82		15	1.34%		1123

## Discussion

In this article, the percentage of acinar predominant tumor is quite high (64.6%) and the percentage of STAS (4.4%) or vascular invasion (1.6%) is exceptionally low compared with those reported in the literature. There was due to the widely application of low-dose CT screening for lung cancer, large amount of lung sub-solid nodules was identified and surgically resected recent years. These cases enrolled in this article were mostly slow-growing lung sub-solid nodules of non-smokers. However, we believe the composition of these enrolled cases may properly represent the early evolution from lung pre-invasive to invasive adenocarcinoma.

This study indicated the different PD-L1 expression status in early-stage lung adenocarcinomas. We demonstrated AIS and MIA were almost PD-L1 expression negative. Interestingly, there was a linear increase in PD-L1 positive rate according to the emerging invasiveness in stage IA1–IA3 lung adenocarcinoma. Thus, we believe PD-L1 pathway may be involved in lung cancer evolution from AIS to IAC, and the precise mechanism is worthy of further illustration.

This study also demonstrated that PD-L1 expression was associated with harmful biological behavior in stage IA lung adenocarcinoma. Firstly, both PD-L1 positive rate and PD-L1 strongly positive rate were significantly increased in cases of IASLC lung adenocarcinoma Grade 3. Further analysis indicate PD-L1 expression was much higher in solid subtype dominant adenocarcinoma. These results may indicate that the PD-L1 pathway might promote the progression of invasive lung adenocarcinoma from lepidic dominant to other harmful biological behavior pathological subtypes. Secondly, we found that PD-L1 expression seemed to be associated with STAS and vascular invasion in stage IA lung adenocarcinoma. STAS is regarded as a significant risk factor for recurrence after surgery in patients with stage IA lung adenocarcinoma [16, 17]. These result was in accordance with former later stage studies, in which PD-L1 expression was related to poor pathological subtype and prognosis in stage IA-IV lung cancer [18–21]. Thus, we believe PD-L1 pathway way be further involved to promote the pathological evolution of IAC.

We also analyzed the association of PD-L1 expression rate with driver gene mutation status. Our data indicated that PD-L1 expression does not correlate with *EGFR* mutation. However, PD-L1 expression seemed to be positively correlated with the mutations of *KRAS*, *ALK*, and *ROS-1* genes. However, our result was not in accordance with the former meta-analysis, which indicate there were no correction in PD-L1 expression with *ALK* and *KRAS* mutation [22]. Thus, the associations between genetic mutations and PD-1/PD-L1 pathway activation in early-stage lung cancer require further investigation.

Few attempts have been made with anti-PD-1 and anti-PD-L1 antibodies in stage IA lung adenocarcinoma. Our study indicated ground glass dominant nodules in CT imaging, including AIS and MIA, were almost PD-L1 negative. Our result was similar to those former studies showing that lepidic pattern adenocarcinoma presenting as GGOs exhibited lower PD-L1 expression related to immune regulation than lung adenocarcinoma of solid nodules [23–26]. Thus, PD-1/PD-L1 inhibitors may not effectively treat early-stage lung cancer of glass ground dominant nodules. However, the attempt of PD-1/PD-L1 pathway inhibitor in early-stage lung cancer of solid nodule was worthy in some cases.

The deficiency of this study was that the IHC antibody used was SP-28-8, not the standard antibody SP-22C3; this may cause some misleading findings [27]. The observation bias of pathologists in the judgment of PD-L1 positivity may also result in uncertainty in the findings of our study.

## Conclusion

In conclusion, our study indicated that PD-L1 expression was positively correlated with the emerging invasiveness of early-stage lung adenocarcinoma. Additionally, PD-L1 expression was also positively correlated with poor pathological subtype and poor biological behavior such as IASLC lung adenocarcinoma Grade 3, solid subtype dominant, STAS and vascular invasion status. Our findings suggest that the PD-1/PD-L1 pathway may be involved in the early evolution of stage 0-IA lung adenocarcinoma.

## Fundings

This work was supported by the National Nature Science Foundation of China (31400713, 82170110), the Shanghai Municipal Key Clinical Specialty (shslczdzk02201), the Shanghai Pujiang Program (20PJ1402400), and the Shanghai Engineer & Technology Research Center of Internet of Things for Respiratory Medicine (20DZ2254400). The funding agency played no part in study design, data analysis, interpretation of data, or manuscript preparation.

## Data Availability

The datasets used and analyzed during the current study are available from the corresponding author on reasonable request.

## Abbreviations

PD-L1: Programmed cell death-ligand 1
AIS: Adenocarcinoma in situ
MIA: Minimally invasive adenocarcinoma
IAC: Invasive adenocarcinoma
STAS: Spread through air space
PD‐1: Programmed cell death‐1
IASCL: International Association for the Study of Lung Cancer
ATS: American Thoracic Society
ERS: European Respiratory Society
WHO: World Health Organization
GGO: Ground glass opacity
IHC: Immunohistochemistry
